# Lifestyle Modifications and Breast Cancer Risk

**DOI:** 10.3390/cancers15112870

**Published:** 2023-05-23

**Authors:** Andrea Manni, Karam El-Bayoumy

**Affiliations:** 1 Division of Endocrinology, Diabetes and Metabolism, Penn State College of Medicine, Milton S. Hershey Medical Center, Hershey, PA 17033, USA; 2 Department of Biochemistry & Molecular Biology, Penn State College of Medicine, Milton S. Hershey Medical Center, Hershey, PA 17033, USA; kelbayoumy@pennstatehealth.psu.edu

Lifestyle modifications have been shown to be effective in reducing breast cancer [1,2,3]. According to a report by the World Health Organization, about 30–50% of cancer cases can be prevented by adopting healthy lifestyles [4]. Such interventions are attractive because they are not associated with toxicity but rather health-promoting effects that go beyond solely breast cancer prevention. Among the lifestyle habits, diet and exercise are the major determinants of breast cancer development and progression and are the focus of the five articles published in this Special Issue on *Lifestyle Modifications and Breast Cancer Risk*. Although, overall, all articles highlight the beneficial effects of a healthy diet or enhanced physical activity, they also emphasize the importance of customizing these interventions in order to ensure the maximum benefit.

In a preclinical study utilizing two estrogen-receptor-negative transgenic mouse models, Brane et al. [5] demonstrate the importance of the dietary administration of sulforaphane, a phytochemical found in cruciferous vegetables, during puberty for the prevention of estrogen-receptor-negative (ER−) breast cancer in later life. In contrast, these authors previously reported that such an intervention had no significant effect on tumor incidence or latency when employed during adulthood [6]. These findings reinforce the notion that puberty, a time when female breast tissues undergo proliferation and differentiation, is a critical period to target interventions aimed at reducing the risk of breast cancer later in life.

The concept of precision onco-nutrition, i.e., the use of specific nutrients and dietary factors to enhance antitumor activity, is explored by Thompson et al. [7] in a series of proof-of-concept in vitro studies testing the antitumor action of omega-3 fatty acids (n3FA) against different cell lines representing the common molecular subtypes of breast cancer. The authors report that n3FA differed in anticancer activity, with docosahexaenoic acid (DHA) being more effective than eicosapentaenoic acid (EPA). The data also highlight the importance of DHA metabolism in mediating its antitumor action, as exemplified by the superior antitumor effect of 4-oxo-docosahexaenoic (4-oxo-DHA), a penultimate metabolite of 5-lipoxygenase-mediated DHA metabolism, compared to DHA. Finally, the antitumor effects of DHA and 4-oxo-DHA varied across cell lines, with the triple-negative types being the most susceptible to growth inhibition. These observations may at least partly explain the inconsistent reports in the literature on the tumor-protective effects of n3FA in breast cancer since the above variables are frequently not accounted for, as discussed by us in [8].

The protective effect of exercise against breast cancer may also not be uniform across the board but may differ depending upon the timing of physical activity in life or anthropometric characteristics. In a longitudinal study involving 15,983 women with a median follow-up of 23.2 years, Boraka et al. [9] reported that the reduction in breast cancer risk provided by high physical activity was most pronounced among perimenopausal and postmenopausal women and women with a waist circumference, body fat percentage or BMI in the upper normal and overweight range. In contrast, for premenopausal women, women with obesity or those with a large body composition, high physical activity did not modify breast cancer risk.

Prescribing the minimum recommended amount of physical activity for general health and breast cancer survivors (≥150 min of at least moderate intensity aerobic activity per week [10,11]) is problematic, especially in older, obese and sedentary subjects with co-morbidities. This issue is addressed in the pilot study by Fabian, CJ et al. [12], which supports the feasibility of a partially supervised exercise concomitantly with caloric restriction for obese, postmenopausal sedentary women to improve body composition and breast cancer risk biomarkers. This 24-week program consisted of twice-weekly professional training sessions (only for the first 12 weeks), membership for exercise facilities and behavioral support. If the results are confirmed in a larger study, this short-term intervention might be translatable to the community since it is similar in concept and duration to many third-party-covered rehabilitation programs after major cardiac surgery.

Collectively, the manuscripts in this Special Issue highlight the multiplicity of the cellular mechanisms by which diet and exercise may influence breast cancer risk. They include: (a) alterations in body composition resulting in changes in adipokine profile [12] and possibly hormone signaling [9]; (b) influence on cellular pathways and molecular targets such as PPARɤ, mTOR, and lipogenesis, which affect the balance between cell proliferation and apoptosis [7]; (c) alterations in the long-term expression of cancer-associated genes, including p21, p53, and BRCA2 (5); and (d) epigenetic regulation [5,13]. In this regard, Gillman et al. [13] assessed the methylation of 11 breast-cancer-related genes at baseline, after completing 16 weeks of supervised exercise intervention and six months after the completion of the intervention in a group of 135 women. Although the total amount of exercise completed was not associated with changes in DNA methylation, the authors reported that increases in VO2 max, a marker of physical fitness, were associated with lower levels of methylation of the BRCA1 gene. They also observed that higher levels of exercise during the follow-up period were associated with a lower level of methylation of the tumor suppressor gene GALNT9. These findings suggest that increased exercise or fitness may affect the methylation of some genes that are related to breast cancer.

In summary, all the articles in this Special Issue provide support in favor of promoting a healthy diet and exercise for reducing breast cancer risk. However, they also highlight critical gaps in our knowledge that need to be addressed in future research in order to optimally customize a specific intervention to the appropriate target population.
